# Glomangiomyoma of Uncertain Malignant Potential in the Urinary Bladder: A Case Report

**DOI:** 10.31729/jnma.5388

**Published:** 2021-07-31

**Authors:** Sanat Chalise, Abhimanyu Jha, Prakash Raj Neupane

**Affiliations:** 1 Department of Pathology, Bhaktapur Cancer Hospital, Dudhapati, Bhaktapur, Nepal; 2 Department of Pathology, Tribhuvan University, Institute of Medicine, Teaching Hospital, Maharajgunj, Kathmandu, Nepal; 3 Department of General Surgery, B&B Hospital Private Limited, Gwarko, Lalitpur, Nepal

**Keywords:** *glomus tumor*, *immunohistochemistry*, *urinary bladder*

## Abstract

Glomus tumour typically occurs in subcutaneous tissue but rarely in the viscera! organs. Most glomus tumours are benign but few atypical glomus tumours have been reported. Herein, we report a case of a 44-year-old male who presented with hematuria. Transurethral resection of bladder tumour was done. Microscopic examination showed nests and sheets of tumor around the blood vessels. Spindle cells resembling smooth muscle were also observed. An increase in mitosis was observed. These tumour cells show diffuse and strong cytoplasmic positivity for smooth muscle actin and negative for Pancytokeratin, Desmin, Synaptophysin, Chromogranin, S100, and Cluster of Differentiation 34. Ki-67 index was approximately 5%. To our knowledge, this is the first report of Glomangiomyoma of uncertain malignant potential in the urinary bladder which is considered as an unusual variant of atypical glomus tumor. This case emphasizes the importance of broad differential diagnosis which has to be considered in the urinary bladder mass.

## INTRODUCTION

Glomus tumor arises from the neuromyoarterial cells of the normal glomus apparatus present in the reticular dermis.^1^ Most of these tumours are benign but some with atypical/malignant behavior have been reported.^2^ These tumours are rare in internal organs where normal glomus bodies are sparse or even absent however, they have been reported in extradigital sites such as stomach, colon, mediastinum, rectum, mesentry and liver.^3^ Glomangiomyomas are least common variant of Glomus tumor.^4^ In this report, we present the rare case of glomangiomyoma of uncertain malignant potential in urinary bladder that has not been previously described in the urinary bladder.

## CASE REPORT

A 44-year-old male attended the surgical out patient department with a complain of gross hematuria for one month. The patient didn't report any other urinary symptoms. He didn't take any medications and was non alcoholic as well as non smoker. There was no relevant past history and family history. No abnormal findings were noted on physical examination. On laboratory examination, his hematological and biochemical parameters were within normal limit. Routine urine examination reveals red blood cells in urine.

The sonographic test showed a round to oval hypoechoeic mass of 1.6x1.3x0.7cm in the urinary bladder. The clinical diagnosis of Urothelial neoplasm was made. Then, the patient was planned for cystourethroscopic evaluation and biopsy. On cystourethroscopy, 1.5cm polypoidal mass was observed in the right lateral wall of urinary bladder. The mass has smooth surface and was firmly attached to the urinary bladder wall. TURBT was done and the mass was sent for histopathological examination.

**Figure 1:**
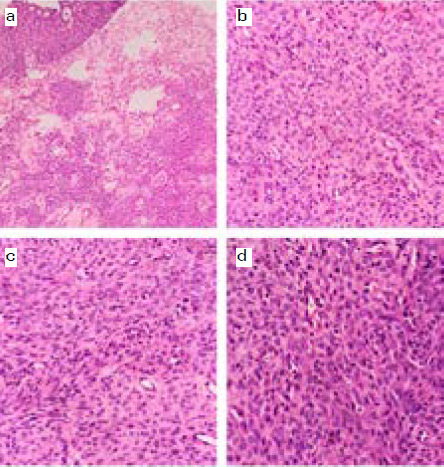
Routine histology with hematoxylin and eosin (H&E) stain a) Proliferation of tumor cells in the lamina propria clustered around the blood vessels. The overlying urothelium is unremarkable. (H&E X50). b) Nests of tumor cells exhibiting monomorphic and bland nuclei with eosinophilic cytoplasm. (H&E X200). c) Transition of tumor cells from oval to spindle shaped resembling smooth muscle. (H&E X200). d) Three mitosis in a single high power field. (H&E X200).

On gross pathological examination, the biopsy consists of four pieces of grey white soft tissue size ranging from 0.2 to 0.5cm and weighing 5gm. Microscopic examination revealed diffuse sheets and nests of tumor clustered around thin, dilated and branching blood vessels (Figure 1a). The cells are monotonous with eosinophilic cytoplasm and round to oval nucleus (Figure 1b). Focally the transition from oval to spindle cells resembling smooth muscle was also observed (Figure 1c). Mitosis was seen comprises of 5/10HPFs however atypical mitosis, necrosis and nuclear pleomorphism was not identified (Figure 1d). The overlying urothelium was unremarkable.

Subsequently, immunohistochemical examination was performed (Table 1).

**Table 1:** Immunohistochemistry results.

Antibody	Clone	Source	Dilution	Reaction
SMA	1A4	Dako	1:500	Diffuse Positive-strong, cytoplasmic.
Pancyto-keratin	AE1/AE3	Dako	1:400	Negative in tumor cells
S-100	Polyclonal	Dako	1:1000	Negative in tumor cells
Desmin	D33	Dako	1:200	Negative in tumor cells
SYP	DAK-SYN-AP	Dako	1:200	Negative in tumor cells
Chromogranin	DAK-A3	Dako	1:400	Negative in tumor cells
Ki-67	MIB-1	Dako	1:100	Approximately 5%
CD34	QBEnd10	Dako	1:50	Capillary endothelial cells are positive and tumor cells are negative

**Figure 2:**
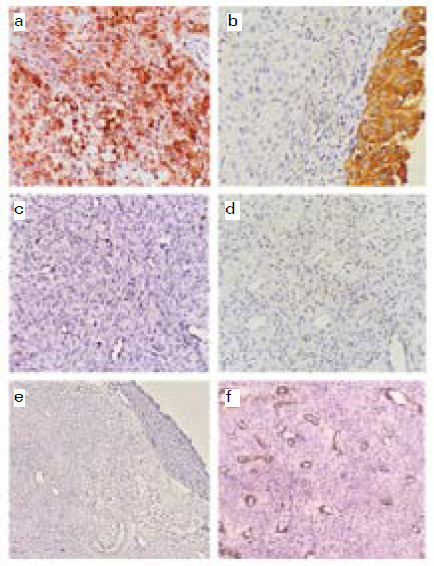
Immunohistochemical findings a) Tumour cells show strong positivity for SMA (Original magnification X400) b) Neoplastic cells did not express AE1/AE3 (Original magnification X200) c) Nuclear staining of Ki-67 showing positivity of about 5%) (Original magnification X400) d) Neoplastic cells did not express Desmin (Original magnification X200) e) Neoplastic cells did not express SYP (Original magnification X50) f) Capillary endothelial cells are positive and tumour cells are negative for CD34 (Original magnification X50).

Capillary endothelial cells are positive and tumor cells are negativeTumor cells were positive for smooth muscle actin (SMA) (Figure 2a). Tumor cells were negative for Pancytokeratin (AE1/AE3) (Figure 2b). Cell proliferation marker, Ki-67 index was approximately 5% (Figure 2c). Tumor cells did not express Desmin (Figure 2d), Synaptophysin (SYP) (Figure 2e), Chromogranin and S100. Capillary endothelial cells were positive and tumor cells were negative for CD34 (Figure 2f). Based on constellation of histopathological findings and IHC a final diagnosis of Glomangiomyoma of uncertain malignant potential was made.

The patient was discharged on 4th post-operative day. Regular follow-up for four years revealed no signs of recurrence.

## DISCUSSION

Genitourinary tract Glomus tumour are extremely rare and the majority of cases are reported in cervix, vagina, kidney and renal pelvis.^5^ Glomus tumour are considered as a hamartomatous proliferation of normal glomus cells which are involved in thermal regulation and was first described by Masson in 1924.^6^ Based on histopathology examination, Glomus tumour are classified into typical and atypical.^2^ Typical Glomus tumour are subclassified into solid Glomus tumour, glomangioma and glomangiomyoma depending on the relative proportion of round glomus cells, vascular smooth muscle cells and spindle cells.^6^ Solid Glomus tumours are composed of solid sheets of glomus cells interrupted by vessels of varying size whereas glomangiomas are less well circumscribed and are composed of hemangioma like vascular structure with small clusters of glomus cells in their wall.^2^ Likewise glomangiomyoma resemble to those of solid Glomus tumour and glomangioma however there is gradual transition of round to oval glomus cells to elongated mature smooth muscle cells.^7^ On the other side, Folpe and colleagues based on study of 52 cases suggested the criteria and subclassified atypical Glomus tumour into malignant Glomus tumour (glomangiosarcoma), glomus tumour of uncertain malignant potential (GLUMP), symplastic Glomus tumour and glomangiomatosis.^8^ They establish the criteria based on the fact that metastatic lesions were seen in 38% of tumour fulfilling the criteria of malignant Glomus tumour while metastasis was not seen in others.^8^

Glomus tumour in urinary bladder is exceedingly rare as glomus bodies are typically absent in visceral organ and exact pathogenesis in not well understood.^6^ After extensive search of database, we found only three reported cases of Glomus tumour in the urinary bladder of which one is benign,^2^ one is malignant^9^ and one is Atypical GLUMP^10^ however to the best of our knowledge glomangiomyoma of uncertain malignant potential has not been reported previously.

We diagnosed this case as Glomangiomyoma of uncertain malignant potential based on histomorphology and the criteria proposed by Folpe, et al.^8^ In our case, the tumour is of visceral origin which describes the deep location and has high mitotic activity. The diagnosis has been confirmed with the aid of immunohistochemistry. It is necessary to differentiate Glomus tumour from other tumours such as urothelial carcinoma, hemangiopericytoma, epithelioid leiomyoma, carcinoid tumour and paraganglioma. The nested areas in the lesion suggest urothelial carcinoma however histomorphological features as well as negative IHC stains for AE1/AE3 exclude urothelial carcinoma. The presence of hemangiopericytic (staghorn) vascular pattern in the present case suggest the diagnosis of hemangiopericytoma but strong immunopositivity of SMA in the tumour cells and absence of CD 34 immonostaining in tumour cells in conjunction with the histomorphological features ruled out hemangiopericytoma. Furthermore, in our case the tumour cells were negative for desmin, excluding and epithelioid leiomyoma. The reported case here in was unlikely to be a carcinoid tumour and paraganglioma, since tumour cells lack both neuroendocrine marker (chromogranin and SYP) as well as S100.

Conclusively, we can say that this is an exceptional case presented as a urinary bladder mass which resembles urothelial neoplasm clinically. Histopathological finding requires confirmation with IHC, which include positivity for SMA to establish the definite diagnosis and help to differentiate it from other mimickers.
